# Post-Treatment Neutrophil and Lymphocyte Counts Predict Progression-Free Survival Following First-Line Chemotherapy in Hodgkin’s Lymphoma

**DOI:** 10.3390/hematolrep15010012

**Published:** 2023-02-10

**Authors:** Grace Fangmin Tan, Siting Goh, Esther Wei Yin Chang, Ya Hwee Tan, Jianbang Chiang, Valerie Shiwen Yang, Eileen Yi Ling Poon, Nagavalli Somasundaram, Mohamad Farid Bin Harunal Rashid, Miriam Tao, Soon Thye Lim, Choon Kiat Ong, Jason Yongsheng Chan

**Affiliations:** 1 Singhealth Internal Medicine Residency, Singapore General Hospital, Singapore 168753, Singapore; 2 Division of Medical Oncology, National Cancer Centre Singapore, Singapore 169610, Singapore; 3 SingHealth Duke-NUS Blood Cancer Centre, Singapore 168753, Singapore; 4 Institute of Molecular and Cell Biology, Singapore 138673, Singapore; 5 Oncology Academic Clinical Program, Duke-NUS Medical School, Singapore 169857, Singapore; 6 Lymphoma Genomic Translational Research Laboratory, Cellular and Molecular Research, National Cancer Centre Singapore, Singapore 169610, Singapore; 7 Cancer and Stem Cell Biology, Duke-NUS Graduate Medical School, Singapore 169857, Singapore; 8 Cancer Discovery Hub, National Cancer Centre Singapore, Singapore 169610, Singapore

**Keywords:** Hodgkin’s lymphoma, ABVD, lymphopenia, neutropenia, prognosis

## Abstract

Hodgkin’s lymphoma carries an excellent prognosis with modern chemotherapy, but a significant proportion of patients remain refractory to or relapse after first-line treatment. Immunological changes post-treatment, such as chemotherapy-induced neutropenia (CIN) or lymphopenia, have shown prognostic significance in multiple tumor types. Our study aims to investigate the prognostic value of immunologic changes in Hodgkin’s lymphoma by examining the post-treatment lymphocyte count (pALC), neutrophil count (pANC) and the neutrophil-lymphocyte ratio (pNLR). Patients treated for classical Hodgkin’s lymphoma at the National Cancer Centre Singapore using ABVD-based regimens were retrospectively analyzed. An optimal cut-off value for high pANC, low pALC and high pNLR in predicting progression-free survival was determined by receiver operating curve analysis. Survival analysis was performed using the Kaplan–Meier method and multivariable Cox proportional models. Overall OS and PFS were excellent, with a 5-year OS of 99.2% and a 5-year PFS of 88.2%. Poorer PFS was associated with high pANC (HR 2.99, *p* = 0.0392), low pALC (HR 3.95, *p* = 0.0038) and high pNLR (*p* = 0.0078). In conclusion, high pANC, low pALC and high pNLR confer a poorer prognosis for Hodgkin’s lymphoma. Future studies should evaluate the potential of improving treatment outcomes by the adjustment of chemotherapy dose intensity based on post-treatment blood counts.

## 1. Introduction

Since the establishment of effective combination chemotherapy, Hodgkin’s lymphoma has been a highly treatable disease with an excellent prognosis. However, a significant proportion of patients remain refractory to treatment or eventually relapse [1], necessitating salvage therapies with a view to autologous stem cell transplant in eligible patients. Approximately 30–50% of this group of patients fail to achieve complete remission after second-line treatment, resulting in poor outcomes [2]. As such, there remains an unmet need to predict treatment failure and reduce rates of relapse. Biologically, classical Hodgkin’s lymphoma is a unique disease—malignant Reed–Sternberg (RS) cells represent only approximately 1% of the tissue compartment of affected lymph nodes, with the majority of cells comprising a mixed infiltrate of benign leukocytes. These leukocytes are thought to be recruited by the RS cells, which in turn sustain their survival [3]. Consequently, the tumor microenvironment and host immune response may play a critical role in the pathobiology of Hodgkin’s lymphoma.

Inflammation and the host immune response play a vital role in the pathogenesis of cancers. Inflammation promotes processes ranging from tumorigenesis to metastasis through the release of cytokines, proteases and pro-angiogenic factors [4]. There is increasing evidence across multiple tumor types that both tumor microenvironment composition and markers of systemic inflammation correlate with patient outcomes. Notably, lymphopenia at diagnosis correlates with poorer overall survival in breast and colorectal cancers [5], as well as diffuse large B cell lymphoma [6,7]. A higher neutrophil-lymphocyte ratio (NLR) at diagnosis correlates with poorer prognosis in many solid tumors such as gastric cancer [8], non-small cell lung cancer [9], soft tissue sarcoma [10] and others. Specifically, in classical Hodgkin’s lymphoma, a previous study has shown that high NLR was an independent prognostic factor of poor overall survival [11].

In addition to baseline leukocyte counts, improved survival with mild neutropenia has been observed across multiple tumor types [12,13]. Conversely, while lymphopenia is well established to be a poor prognostic factor in many malignancies, including that of advanced Hodgkin’s lymphoma (as part of the International Prognostic Score) [14], the prognostic implications of post-chemotherapy lymphopenia are less well-studied. Severe chemoradiation or radiotherapy-induced lymphopenia seems to confer poorer prognosis in tumor types such as esophageal [15,16] and lung cancer [17], but few studies have focused on lymphopenia in the context of chemotherapy alone.

Therefore, this study aims to investigate the impact of the post-treatment absolute neutrophil count (pANC) and absolute lymphocyte count (pALC) following first-line ABVD-based chemotherapy on progression-free survival in Hodgkin’s lymphoma. We also explore the prognostic significance of the post-treatment neutrophil-lymphocyte ratio (pNLR), derived from pANC and pALC, as a potential unified predictor of PFS.

## 2. Materials and Methods

### 2.1. Study Cohort

**Figure 1:**
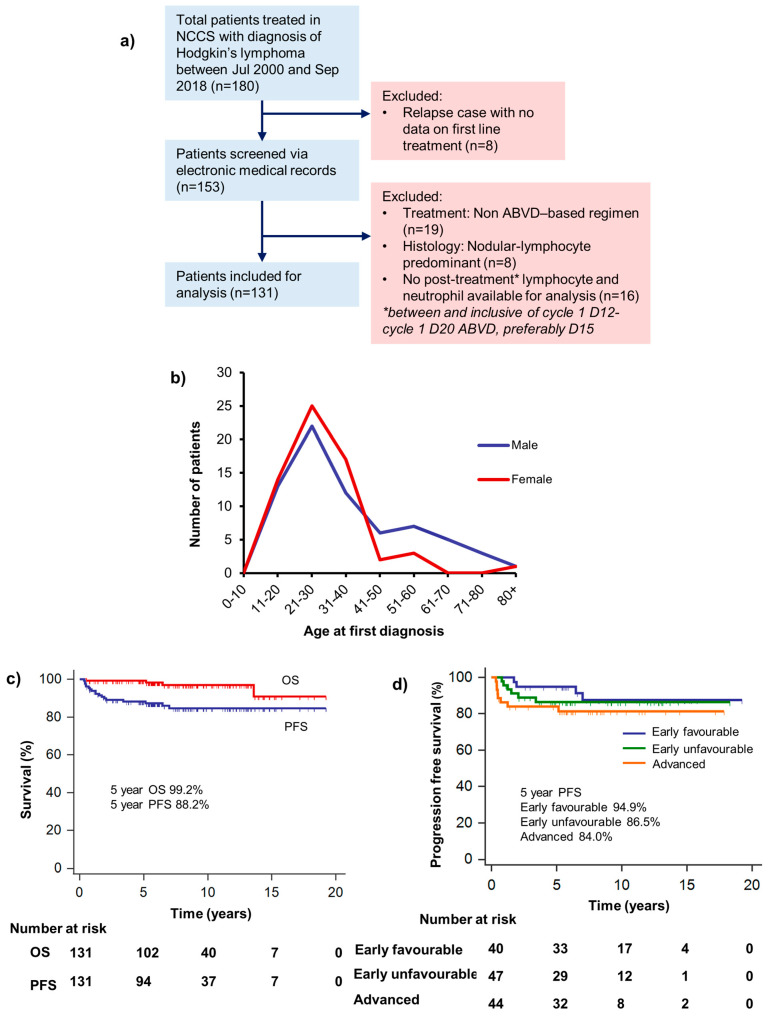
(**a**) Consort diagram illustrating our study design and patient selection process. (**b**) Age distribution of male and female patients in our cohort at first diagnosis. Incidence of Hodgkin’s was bimodal in our patient population, similar to previous studies. (**c**) Overall survival and progression-free survival in our cohort of patients with Hodgkins’ lymphoma. (**d**) Progression-free survival stratified by German Hodgkin Study Group (GHSG) stage. Note that PFS for all stages is excellent, with 81.3% 10-year PFS for GHSG advanced Hodgkin’s’ patients.

Medical records of all patients with a diagnosis of Hodgkin’s lymphoma seen at the National Cancer Centre Singapore between July 2000 to September 2018 were retrospectively reviewed (*n* = 180), of which records of first-line therapy were available for 172 patients. Patients who did not receive an ABVD-based treatment regimen, had a diagnosis of nodular-lymphocyte predominant Hodgkin’s lymphoma or had indeterminate histology were excluded from the analysis. Of the remaining subjects, a total of 131 patients who had available peripheral blood neutrophil (ANC) and lymphocyte counts (ALC) at the time of diagnosis and between days 10 and 20 from the first cycle of chemotherapy were included for analysis (Figure 1a). None of the included patients had evidence of an infectious process or rheumatological disorder. Patients received a median of 6 cycles of ABVD (range 2 to 8). Consolidation radiation therapy was administered to 89 patients (67.9%).

Median follow-up was 6.6 years. Clinical and pathological information available included age, sex, ethnicity, Eastern Cooperative Oncology Group (ECOG) performance status, stage, histological subtype, presence of bulky disease, presence of B symptoms, bone marrow involvement and presence of raised lactate dehydrogenase (LDH) levels, as summarized in Table 1. The sex, age and ethnicity of the subjects were verified against their National Registry Identification Cards. ALC and ANC were collected from electronic medical records at the time of diagnosis, as well as between days 10 and 20 from the first cycle of chemotherapy, the latter designated as pALC and pANC, respectively. For patients with multiple ANC and ALC readings available between days 10 and 20, counts nearest to day 15 were selected for analysis. Bulky disease was defined as a mediastinal mass of more than one-third intra-thoracic diameter on relevant imaging or any mass ≥ 10 cm on cross-sectional imaging at diagnosis. Staging was based on the Ann Arbor Staging Classification for Hodgkin’s lymphoma and German Hodgkin Study Group (GHSG) risk group classification systems.

### 2.2. Statistical Analysis

The pNLR was calculated by dividing pANC by pALC taken at the same sitting. Receiver operating characteristic (ROC) curve analysis via the method of DeLong et al. [18] was used to identify the optimal discriminatory cut-off value for pANC, pALC and pNLR as univariable predictors of PFS. Spearman rank correlation was used to compare pre- and post-treatment blood indices. Comparisons of the frequencies of categorical variables were performed using Pearson’s Chi-squared tests. The primary and secondary survival endpoints of interest in this study are PFS and OS, respectively. For analyses of OS, survival was measured from the date of diagnosis until the date of death from any cause or was censored at the date of the last follow-up for survivors. PFS was defined as the time elapsed from the date of diagnosis until the date of progression, relapse or death from any cause. Kaplan–Meier survival curves were plotted to estimate actuarial survival and compared using the log-rank test, and factors with *p* < 0.2 were selected for multivariable analysis. Cox proportional hazards regression was used to calculate hazard ratios (HR) with corresponding 95% confidence intervals (95% CI) of mortality. A multivariable Cox regression model via a stepwise procedure was used to test for independence of selected (*p* < 0.2) factors identified on univariate analysis. All statistical evaluations were made assuming a two-sided test with a significance level of 0.05 unless otherwise stated. All tests were performed using MedCalc statistical software for Windows version 19.6 (MedCalc Software, Ostend, Belgium).

## 3. Results

### 3.1. Patient Characteristics

In our cohort, the median age at diagnosis was 29 years (range 15 to 81) with a bimodal age distribution, as illustrated in Figure 1b. There were 69 (52.7%) male patients and 62 (47.3%) female patients. GHSG stage was early favorable in 40 (30.5%), early unfavorable in 47 (35.9%) and advanced in 44 (33.6%). The commonest histological subtype encountered was nodular sclerosing (*n* = 100, 76.3%), followed by mixed cellularity (*n* = 21, 16.0%), lymphocyte rich (6, 4.6%) and classical non-otherwise specified (NOS) (*n* = 4, 3.1%). At diagnosis, bulky disease was present in 46 patients (35.1%), while B symptoms were present in 24 patients (18.3%), and raised lactate dehydrogenase (LDH) was present in 64 (48.9%) of patients (Table 1). At the time of analysis, 11.5% of patients had relapsed or progressed, and 3.1% of patients had died. Overall OS and PFS were excellent, with a 5-year OS of 99.2% (95% CI 97.7% to 100%) and a 5-year PFS of 88.2% (95% CI 82.6% to 93.8%) (Figure 1c,d).

**Figure 2:**
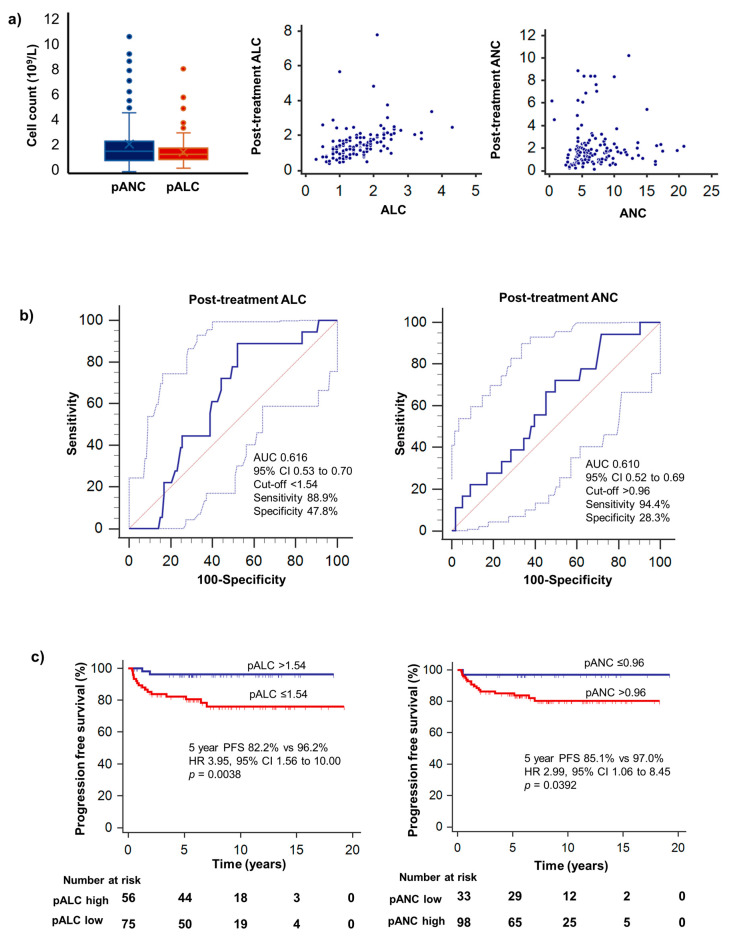
(**a**) Distribution of pALC and pANC in our cohort, and scatter plot illustrating correlation of pre- and post-treatment ALC and ANC. (**b**) Optimal cut-off values for stratification of low pALC (≤1.54) and high pANC (>0.96) were determined via receiver operating curve analysis. Dotted lines represent 95% confidence interval for sensitivity and specificity. (**c**) PFS stratified by pALC and pANC. High pALC (>1.54) and low pANC (≤0.96) were correlated with better progression-free survival.

The pANC (median: 1.64, range 0.11 to 10.2), pALC (median: 1.42, range 0.39 to 7.8) and pNLR (median: 1.14, range 0.13 to 13.1) varied widely throughout the cohort. Pre and post-treatment ANC, ALC and NLR were compared. ALC was significantly correlated with pALC (*p* < 0.0001, rho = 0.534, 95% CI 0.400–0.647), and NLR significantly correlated with pNLR (*p* = 0.0001, rho = 0.329, 95% CI 0.167–0.474). ANC did not significantly correlate with pANC (*p* = 0.1347, rho = 0.131, 95% CI 0.0411–0.296) (Figure 2a). Patients were categorized according to levels of pANC, pALC and pNLR using optimized cut-offs to predict PFS as derived from ROC curve analysis (>0.96, ≤1.54 and >0.68, respectively), with 98 (74.8%) patients considered pANC high, 75 (57.3%) patients considered pALC low, and 97 (74.0%) patients considered pNLR high. The areas under the curve (AUC) for ROC analysis were 0.610, 0.616 and 0.646 for pANC, pALC and pNLR, respectively (Figure 2b).

### 3.2. Clinicopathological Correlation

In a multivariable analysis, low pALC was not significantly associated with sex, age, ethnicity, ECOG performance status, GHSG stage, histological subtype, presence of bulky disease, B symptoms or raised LDH. Conversely, high pANC was associated with non-Chinese ethnicity (*p* = 0.0176) and female sex (*p* = 0.0176), but not with any of the other abovementioned variables. Multivariable analysis of the same clinicopathological parameters was carried out for leukocyte counts before treatment. High ANC (>9.8) at diagnosis was associated with raised LDH (*p* = 0.002), while low ALC at diagnosis (≤1.1) was associated with the presence of bulky disease (*p* = 0.0173) and ECOG ≥ 1 (*p* = 0.0288). Cut-offs for high ANC and low ALC at diagnosis were determined by ROC analysis.

### 3.3. Survival Analysis

**Figure 3:**
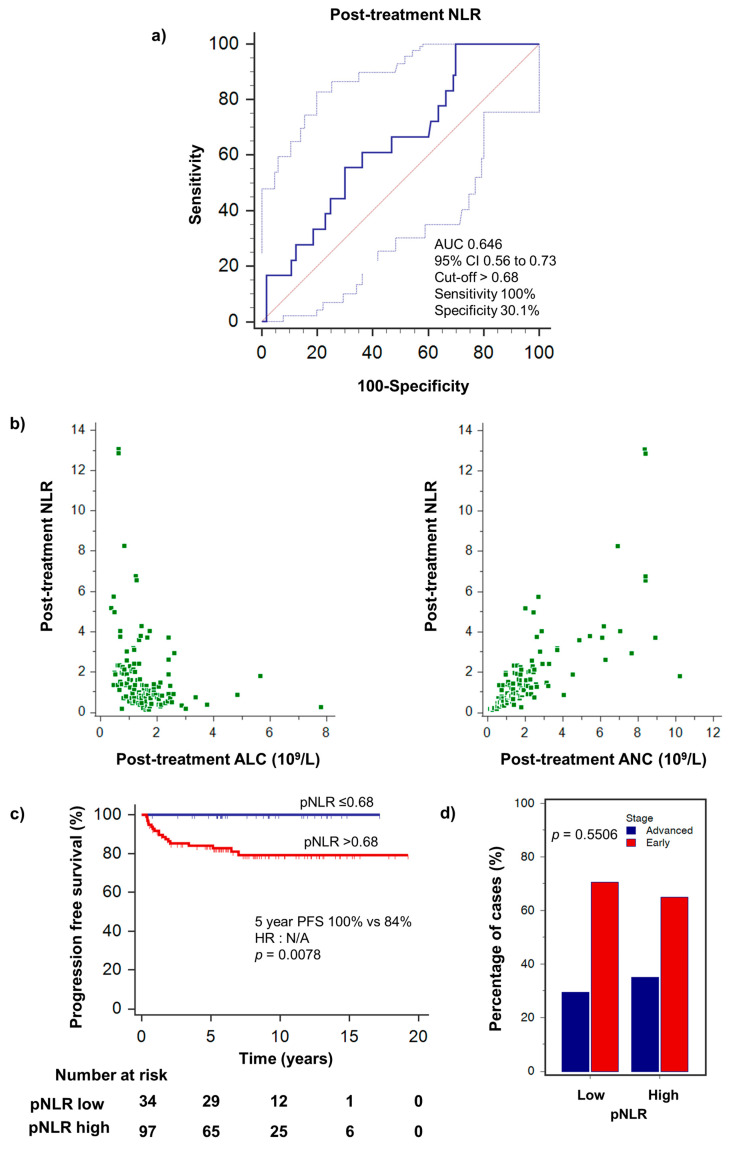
(**a**) Optimal cut-off value for high pNLR (>0.68) was determined by receiver operating curve analysis. (**b**) Correlation of post-treatment NLR with post-treatment ALC (rho = −0.474, *p* < 0.0001) and ANC (rho = 0.804, *p* < 0.0001). (**c**) PFS stratified by high/low pNLR. Low pNLR ≤ 0.68 was significantly associated with better progression-free survival. Remarkably, PFS was 100% in the low pNLR cohort. (**d**) pNLR did not correlate with disease stage.

Poorer PFS was significantly associated with high pANC (HR 2.99, 95% CI 1.06–8.45, *p* = 0.0392) and low pALC (HR 3.95, 95% CI 1.56–10.00, *p* = 0.0038) (Table 2). The 5-year PFS for patients with low pALC was 82.2% as compared to 96.2% for high pALC and 97.0% for patients with low pANC vs. 85.1% for patients high pANC (Figure 2c). In multivariable analysis, low pALC remained an independent predictor of poorer PFS (HR 6.80, 95% CI 1.56–29.59, *p* = 0.0107) (Table 3). We demonstrated that post-treatment NLR was correlated with both neutrophil counts (rho = 0.804, *p* < 0.0001) and lymphocyte counts (rho = −0.474, *p* < 0.0001). High pNLR was also significantly associated with poorer PFS (*p* = 0.0078). pNLR did not correlate with the disease stage (*p* = 0.5506) (Figure 3). Remarkably, in our patient population, no patients with low pNLR experienced relapse/progression during the period of follow-up. Other prognostic factors such as ECOG performance status, GHSG stage and raised LDH did not significantly correlate with PFS.

Similar trends were observed for pANC, pALC and pNLR when subgrouped by disease stage (Supplementary Figure S1). We performed an additional analysis to evaluate the prognostic impact of ANC, ALC and NLR at diagnosis. A trend was observed that high ANC, low ALC and high NLR were all associated with worse PFS, though they did not reach statistical significance (Supplementary Figure S2). Correlation with OS was not performed since only 4 patients had died at the time of analysis.

## 4. Discussion

In this study, we investigated the prognostic implications of neutrophil and lymphocyte counts following first-line ABVD-based chemotherapy (termed pANC and pALC, respectively) on survival outcomes. The characteristics of our patient cohort were similar to previous studies in developed countries [19]. Our study population had a bimodal age distribution with the highest incidence in young adults, and there was an equal sex distribution. In keeping with our hypothesis, a high pANC, low pALC, and high pNLR conferred significantly poorer PFS.

Chemotherapy-induced neutropenia (CIN) is conventionally regarded as a potentially dangerous side effect of cytotoxic chemotherapy, so it is interesting that multiple studies have recently demonstrated survival benefits in patients with CIN. In our study, we showed that pANC of less than 0.96 × 10^9^/L (entailing grade 3 neutropenia) may potentially confer PFS benefit following ABVD-based chemotherapy. The exact mechanisms remain unclear, though we postulate that the presence of CIN may imply inadequate cytotoxic efficacy. In contemporary practice, chemotherapy dosing is primarily guided by body surface area. While this is convenient, it is known that this convention fails to account for inter-patient differences in metabolism and drug clearance [20]. For example, a large range of genetic polymorphisms affects drug metabolism pathways such as those involving CYP enzymes and purine/pyrimidine metabolism, in turn affecting the pharmacokinetics of a range of chemotherapy agents [21]. Low pANC in Hodgkin’s lymphoma may thus serve as a more personalised marker of the adequacy of chemotherapy dosing and treatment efficacy. This is similar to what has been suggested for other tumor types, such as pancreatic cancer [22], breast cancer [12], and serous ovarian cancer [13], where a similar trend of survival improvement with CIN has been observed. The beneficial effect of CIN may also derive from the direct effect of neutrophils in malignancy. In recent literature, neutrophils have been shown to promote tumor initiation and growth through the induction of angiogenesis, proliferation and tumor infiltration [4]. Neutrophils may also exert an immunosuppressive effect through the expression of the immune checkpoint ligand PD-L1 [23]. Future studies which seek to better understand the pro-oncogenic effects of neutrophils will be warranted.

Lymphopenia at diagnosis is an established poor prognostic factor for advanced Hodgkin’s lymphoma [14], but the prognostic implications of chemotherapy-induced lymphopenia are less clear. It has been postulated that lymphopenia reflects an underlying immunosuppressed state or may result from tumor-induced lymphocyte destruction and alterations in immune homeostasis in cancer [7]. Lymphopenia also appears to drive the preferential proliferation of regulatory T cells, which could hinder antitumor immune activity [24]. Chemotherapy-induced lymphopenia has been recognized in previous studies, with various effects on B and T cell subpopulations [25,26]. In previous studies on patients with esophageal cancer, grade 4 lymphopenia at the nadir after chemoradiotherapy (CRT) correlated with significantly poorer OS and RFS [15,16]. Interestingly, recovery from lymphopenia did not improve prognosis, and it was hypothesized that lymphopenia recovery might not fully reflect post-treatment changes in lymphocyte subtype and lymphocyte diversity, both of which are critical to normal immune and antitumor activity. Similar trends toward poorer survival with treatment-induced lymphopenia have been noted in cervical cancer and other solid tumors [27,28].

The findings of our study may imply opportunities for the development of novel strategies in patient management and follow-up. In particular, novel therapies such as IL-7 immunotherapy have been previously explored in animal models to enhance lymphocyte recovery after treatment-induced lymphopenia [29,30]. Another potential strategy includes the reinfusion of autologous lymphocytes combined with GM-CSF to aid lymphocyte recovery after chemotherapy [31]. As mentioned, pANC levels may guide the dose adjustment of chemotherapy and potentially serve as an adjunct measure to the interim PET/CT scan when considering patients for escalation or de-escalation of treatment regimens. In addition, since low pANC has a potential PFS advantage, this also perhaps suggests against the prophylactic use of G-CSF without a compelling reason to do so.

Our study is limited by its retrospective nature and relatively small cohort size, with a low event rate of disease progression or relapse. Post-treatment lymphocyte and neutrophil counts were examined only at a single time point, and the relative changes in lymphocyte subtypes after chemotherapy were not known. The optimal cut-off values remain to be determined in larger prospective studies. PET/CT data were also unavailable for the majority of patients.

In conclusion, pANC and pALC following first-line ABVD-based chemotherapy predict PFS in patients with Hodgkin’s lymphoma. Future studies are required to validate these results and investigate their implications for clinical intervention.

## Figures and Tables

**Table 1:** Patient characteristics.

Characteristic	Number (%)
Total	131 (100%)
Age	
Median age (years)	29 (Range: 15–81)
Sex	
Male	69 (52.7%)
Female	62 (47.3%)
Ethnicity	
Chinese	85 (64.9%)
Malay	20 (15.3%)
Indian/Pakistani/Sri Lankan	17 (13.0%)
Other	9 (6.9%)
ECOG performance status	Can be
0	85 (64.9%)
≥1	46 (35.1%)
Ann Arbor stage	
I	24(18.3%)
II	68(51.9%)
III	20 (15.3%)
IV	19 (14.5%)
GHSG stage	
Early (87)	
Favorable	40 (30.5%)
Unfavorable	47 (35.9%)
Advanced (44)	
Stage IIBE/IIBX	5 (3.8%)
Stage III	20 (15.3%)
Stage IV	19 (14.5%)
Histological subtype	
Nodular sclerosis	100 (76.3%)
Mixed cellularity	21 (16.0%)
Lymphocyte rich	6 (4.6%)
Classical (NOS)	4 (3.1%)
Bulky disease	
Present	46 (35.1%)
Absent	85 (64.9%)
B symptoms	
Present	24 (18.3%)
Absent	107 (81.7%)
Raised LDH	
Present	64 (48.9%)
Absent	67 (51.1%)
Bone marrow	
Involved	13 (9.9%)
Not involved	118 (90.1%)

**Table 2:** Univariate survival analysis.

Characteristic	Progression-Free Survival	
	HR (95% CI)	*p*
Age at diagnosis	1.0073 (0.9791–1.0363)	0.6173
Ethnicity (Chinese vs. non-Chinese)	0.7537 (0.2811–2.0203)	0.5740
Sex (male vs. female)	0.7019 (0.2779–1.7729)	0.4540
B symptoms (present vs. absent)	2.3562 (0.6712–8.2713)	0.1810
Bulky disease (present vs. absent)	1.5425 (0.5837–4.0764)	0.3821
ECOG performance status (0 vs. ≥ 1)	0.5104 (0.1962–1.3279)	0.1680
GHSG stage (advanced vs. early)	1.7639 (0.6552–4.7492)	0.2614
Histological subtype (mixed cellular vs. other)	0.8811 (0.2414–3.2160)	0.8480
pALC (low vs. high)	3.9471 (1.5580–9.9995)	0.0038
pANC (high vs. low)	2.9870 (1.0559–8.4504)	0.0392
pNLR (high vs. low)	N/A 5-year PFS 84% vs. 100%	0.0078
Raised LDH (present vs. absent)	1.7852 (0.7066–4.5105)	0.2204

**Table 3:** Cox proportional hazard regression analysis (multivariable analysis).

Characteristic	Progression-Free Survival	
	HR (95% CI)	*p*
pANC (high vs. low)	6.978 (0.927–52.356)	0.0592
pALC (low vs. high)	6.983 (1.604–30.395)	0.0096

Number of events = 19; factors included (*p* < 0.2): B symptoms, ECOG performance status, pALC, pANC.

## Data Availability

The original datasets analyzed during the current study are available from the corresponding author upon reasonable request.

## Supplementary Materials

The following supporting information can be downloaded at: https://www.mdpi.com/article/10.3390/hematolrep15010012/s1, Figure S1: Prognostic significance of pANC, pALC and pNLR subgrouped by stage; Figure S2: Prognostic significance of ANC, ALC and NLR at diagnosis
