# Supplementary material for: Post-Treatment Neutrophil and Lymphocyte Counts Predict Progression-Free Survival Following First-Line Chemotherapy in Hodgkin’s Lymphoma
Source: Hematol Rep. 2023 Feb 10;15(1):108–18. doi: 10.3390/hematolrep15010012 (PMC9944084; doi:10.3390/hematolrep15010012)
Supplement: Supplementary file 1 [file hematolrep-15-00012-s001.zip › hematolrep-2076196-supplementary.pdf]

Supplement to: Post-treatment neutrophil and lymphocyte counts predict progression free survival following first-line chemotherapy in Hodgkin's lymphoma

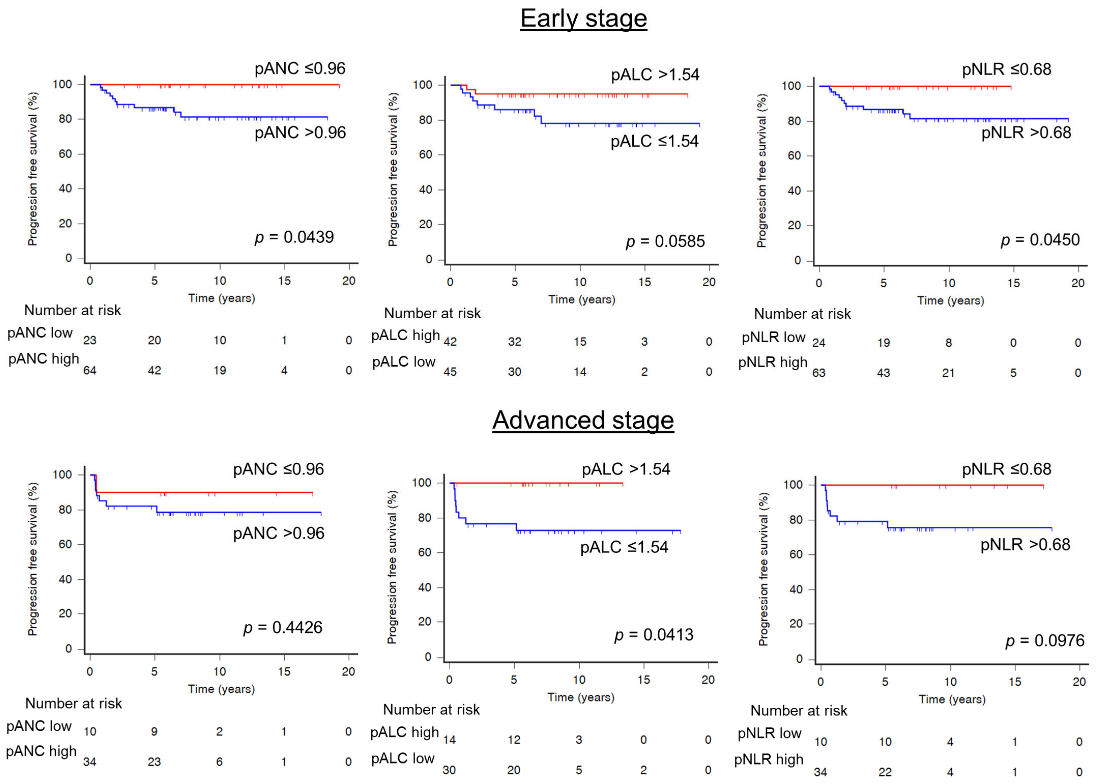

Figure S1. Prognostic significance of pANC, pALC and pNLR subgrouped by stage.

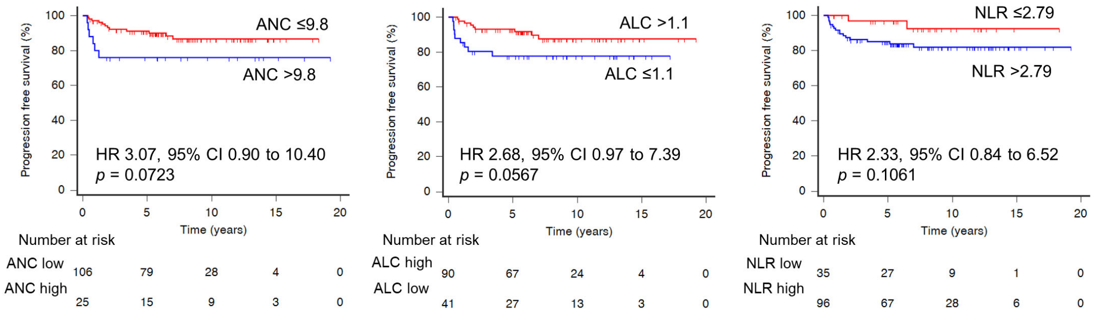

Figure S2. Prognostic significance of ANC, ALC and NLR at diagnosis.
